# Cellular Specificity of NF-κB Function in the Nervous System

**DOI:** 10.3389/fimmu.2019.01043

**Published:** 2019-05-09

**Authors:** Erica C. Dresselhaus, Mollie K. Meffert

**Affiliations:** Department of Biological Chemistry and Solomon H. Snyder Department of Neuroscience, Johns Hopkins University School of Medicine, Baltimore, MD, United States

**Keywords:** NF-κB, gene expression, Plasticity, Neurons, transcription, synapse, glia, central nervous system (CNS)

## Abstract

Nuclear Factor Kappa B (NF-κB) is a ubiquitously expressed transcription factor with key functions in a wide array of biological systems. While the role of NF-κB in processes, such as host immunity and oncogenesis has been more clearly defined, an understanding of the basic functions of NF-κB in the nervous system has lagged behind. The vast cell-type heterogeneity within the central nervous system (CNS) and the interplay between cell-type specific roles of NF-κB contributes to the complexity of understanding NF-κB functions in the brain. In this review, we will focus on the emerging understanding of cell-autonomous regulation of NF-κB signaling as well as the non-cell-autonomous functional impacts of NF-κB activation in the mammalian nervous system. We will focus on recent work which is unlocking the pleiotropic roles of NF-κB in neurons and glial cells (including astrocytes and microglia). Normal physiology as well as disorders of the CNS in which NF-κB signaling has been implicated will be discussed with reference to the lens of cell-type specific responses.

## Background

Mammalian NF-κB functions as a dimer composed of five potential Rel/NF-κB family subunits that can be divided into two classes. Rel A (p65), c-Rel, and Rel B are synthesized as mature proteins which contain transcription transactivation domains (TADs). The remaining subunits, p50 and p52, are post-translationally cleaved from the precursor proteins p105 (NFKB1) and p100 (NFKB2), respectively, and lack TADs (1). NF-κB subunits can hetero- or homo-dimerize; while all subunits have been reported to be expressed in different brain cell types, the p50:p65 dimer appears widely abundant to date. In unstimulated conditions, NF-κB dimers are held latent mainly in the cytoplasm by binding to a set of proteins from the “inhibitor of kB” (IκB) family. Following cellular stimulation by growth factors, excitatory synaptic transmission, immune-modulatory factors, or other activators, the IκB inhibitors undergo post-translational modification and ultimately degradation through well-studied steps which have been reviewed in detail elsewhere (2). These steps may be broadly categorized into either an alternative pathway or the “cannonical pathway” (CP) in which IκB phosphorylation is mediated by the IκB kinase complex (IKK). In the CP, NF-κB activation occurs when incoming cellular stimuli connect to signaling assemblies mediating IKK activation. IKK activation leads to IκB inhibitor phosphorylation on critical serine residues, followed by ubiquitination and proteasomal degradation, which frees the NF-κB dimer to undergo stable translocation into the cellular nucleus and regulate transcription of genes containing consensus κB binding sites in the DNA of their enhancers or promoters. NF-κB-mediated transcription has also been reported to be enhanced through phosphorylation of the Rel proteins (3).

Studies in the nervous system have assessed several aspects of the pathway as proxies to monitor NF-κB activation, including accumulation of Rel proteins in the nucleus and Rel phosphorylation, as well as the classical reduction in IκB protein levels and accompanying induction of NF-κB-DNA binding activity (e.g., by electromobility shift assay, EMSA). Multiple approaches have also been used to test roles for NF-κB in molecular and behavioral studies of brain function. Mutation of the serine residues critical for IκBα phosphorylation and degradation has been used to engineer a dominant-negative “super-repressor” (DN-IκB) transgene that exerts blockade of the NF-κB activation pathway (4), and constitutively active variants of IKK (CA-IKK) have been used to produce genetically controlled NF-κB pathway activation. Viral expression constructs and mouse lines enabling selective expression of the DN-IκB and CA-IKK have also been effective tools in dissecting cell-type specific roles of NF-κB in the CNS. Mouse lines engineered to allow site-specific recombination using Cre-lox systems should become particularly useful in allowing neuroscientists to define endogenous gene function for members of the Rel family in discrete brain regions, cell types, and developmental periods. In the following sections, we will review studies which incorporate multiple approaches to define our current understanding of NF-κB function in the mammalian nervous system during both health and disease, with a particular focus on the complexity of cell-type and subunit-specific roles.

### Normal Physiology

The heterogeneous cellular composition of the brain has presented a major challenge to understanding the effects of NF-κB pathway activation in single cells as well as its role in cognitive function of the brain as a whole. Comparative studies of NF-κB in the many rare cell types of the CNS remains uncharted territory. However, to appreciate the overall impacts of signaling through the NF-κB pathway on brain health and function it is nonetheless instructive to consider more general assessments of activation and function in neurons vs. glia. Effective promoters for driving cell-type selective manipulations of the NF-κB signaling pathway exist for neurons but have until very recently hampered studies of glial-specific roles, as discussed below. Partially due to this limitation, more of the existing evidence for discrete cell-type and subunit-specific roles of NF-κB activation has emerged from selective pathway manipulations in neurons.

## Neurons

There are likely hundreds of different types of neurons within the mammalian CNS, depending upon the classification method (5). Broad neuronal categories in which NF-κB function has been investigated include both excitatory (glutamatergic) and inhibitory (GABAergic) neurons, as well as the neuronal subcompartment of the synapse, the sites where connections between neurons are made. A broad range of stimuli are documented to activate neuronal NF-κB, ranging from well-known inflammatory mediators, to stimuli whose action may participate more selectively in NF-κB signaling in neurons, such as the growth factors neuronal growth factor (NGF) and Brain-derived growth factor (BDNF) and excitatory neurotransmitters (6).

In addition to selective induction pathways, there are also multiple functional readouts of NF-κB activation on both the cellular and behavioral level which appear to be specific for the neuronal population of NF-κB. Three members of the NF-κB family, p50 (7–9), c-Rel (10–12), and p65/RelA (13, 14), as well as IKK (15), have been implicated in regulating cognitive behaviors in mice, including learning and memory. However, most of the studies examining NF-κB subunit-specific effects on behavior have not been carried out in a cell-type selective manner, and have instead used mouse models ubiquitously lacking particular subunits which could have phenotype contributions from loss of NF-κB outside the nervous system, such as immunological deficits. Neuron-selective disruption of NF-κB signaling [by DN-IKK expression predominantly in either forebrain excitatory neurons (16) or GABAergic interneurons] (17) has confirmed essential roles for NF-κB in a variety of assays of synaptic plasticity as well as mammalian cognitive behavior tasks. However, such NF-κB pathway manipulation does not deliver subunit specific information. Several informative reviews on cognitive effects of NF-κB signaling have been published (18–21), and we will not dwell further on this topic here.

Activation of the NF-κB signaling pathway by excitatory neurotransmission and its participation in multiple forms of structural and synaptic plasticity is likely a basis for the function of this transcription factor in cognitive behaviors. Deficits in NF-κB signaling have been shown to produce impairments in *in vitro* assays of long-term plasticity, including long-term potentiation (LTP) (9, 10, 17, 22) and long-term depression (LTD) (12). Activation of the NF-κB pathway in murine excitatory glutamatergic neurons promotes dendritic spine and excitatory synapse formation (23), while diminished NF-κB activity (loss of RelA/p65) reduces dendritic spine size and density as well as miniature excitatory post-synaptic currents (mEPSCs), during developmental periods of synapse formation, or in mature neurons responding to increased synaptic demand (23). Collectively, these effects are consistent with a role for NF-κB in enhancing excitatory synaptic function. While these cell-autonomous effects were observed with manipulation of RelA/p65 in excitatory neurons, it is unknown if they are specific only for the RelA subunit of NF-κB. Diminishing NF-κB activity in inhibitory GABAergic neurons (through selective DN-IκB expression) has been reported to produce a distinct phenotype of diminished inhibitory tone and enhanced excitatory firing (17). NF-κB is also the first transcription factor to be implicated in the feedback mechanisms that regulate the endpoint of homeostatic synaptic plasticity to elevated excitatory activity (24). During the homeostatic response to chronic elevated excitatory activity, NF-κB activation by polo-like kinases (Plks) opposes Plk-mediated degradation of the synapse stabilizing protein, spine-associated RapGTPase-activating protein (SPAR), by transcriptionally upregulating SPAR in hippocampal excitatory neurons *in vitro* and *in vivo*. Neurons which are deficient in NF-κB (RelA/p65) fail to limit homeostatic adjustments in the context of chronic elevated neuronal excitation, producing exaggerated homeostatic reductions in dendritic spines and excitatory synaptic currents (24).

Excitatory neurotransmitters were first demonstrated to activate NF-κB in cultured cerebellar granule neurons (25, 26) and in the developing cerebellum *in vivo*, where NF-κB activation was shown to be sensitive to antagonism of receptors for the glutamate excitatory neurotransmitter (27). The ability of excitatory glutamatergic stimuli to mediate rapid induction of NF-κB through the cannonical pathway appears to be specific to neurons, compared to glial cells. Prolonged (24 hr) exposure of cultured primary astrocytes to glutamate has been reported to generate a toxic, oxidative stress-mediated activation of NF-κB (28), and glutamate stimulation of glioma cell lines can produce secondary non-cannonical NF-κB activation through epidermal growth factor receptor (EGFR) signaling (29). In contrast, rapid activation of neuronal NF-κB downstream of excitatory stimulation occurs predominantly through the NMDA glutamate receptor subtype and L-type voltage sensitive calcium channels in a variety of neurons from distinct brain regions including the cerebellum, hippocampus, and cortex[as reviewed in (30)]. Stimulation through the glutamate metabotropic receptors can also produce neuronal NF-κB activation of p50, p65, and c-Rel subunits as reported through ELISA of area CA1 lysates from hippocampal slices (12).

The gating of NMDA receptors and L-type calcium channels generates the majority of glutamate-mediated calcium influx, which has been implicated in NF-κB activation through the cannonical pathway in neurons from multiple brain regions (13, 26, 31, 32). Consistent with a critical role for calcium elevation in mediating excitatory NF-κB activation, elevating calcium through use of a calcium ionophore was found sufficient to produce IKK activation in studies using hippocampal or striatal neurons (13, 33). Further, calcium-responsive signaling cascades, including transduction through the calcium calmodulin dependent protein kinase II (CaMKII) are critical for glutamate-mediated activation of IKK and NF-κB. CaMKII-dependent activation of the IKK complex appears not to be specific to neurons, however, as CaMKII isoforms have been linked to NF-κB activation in a variety of cells, including T cells, cardiac myocytes, and fibroblasts (34–36). The CaMKIIα isoform has been specifically linked to NF-κB activation where studied in hippocampal neurons and purified retinal ganglion cells (13, 37). CaMKIIα activation downstream of elevated calcium leads directly or indirectly to IKK activation. Activated NF-κB has been shown to undergo dynein-dependent active transport resulting in nuclear accumulation of the transcription factor (38–40).

Calcium elevations in response to excitatory glutamate stimulation are particularly high in the subcompartment of the neuronal synapse. CaMKIIα is also highly enriched in both the synapse and the synaptic region of the post-synaptic density (PSD, a specialized region attached to the post-synaptic membrane opposite presynaptic terminals), where it is well-positioned to respond to incoming calcium signals and plays prominent roles in synaptic plasticity. Like CaMKII, NF-κB, and the IKK and IκB signaling components are also located within synapses. Immunohistochemical evidence of NF-κB in neuronal processes provided a first suggestion of its presence at synapses (26, 41), that was supported by the presence of NF-κB, as p65:p50 dimers, in biochemically isolated synapses (13, 41–43) from wildtype but not p65-deficient mice (13). In excitatory (pyramidal) neurons the post-synaptic side of glutamatergic synapses are located upon small specialized protrusions from the neuronal dendrites, which are known as dendritic spines. NF-κB dimers composed of either p65:p50 or p65:p65 are selectively enriched within dendritic spines in hippocampal pyramidal neurons (23, 44), and are also found in the PSD (45). Analysis of a series of truncation and deletion mutants narrowed the region of the p65 subunit critical for enrichment within dendritic spines to a 30 amino acid section in the mid-region of p65 protein, located between the amino-terminal Rel homology domain (RHD) and the C-terminal transactivation domain (TAD) (44). A p65 minimal mutant lacking spine enrichment but retaining transcriptional activity was selectively deficient in transcriptional responses to stimuli incoming through the excitatory synapses on dendritic spines, in comparison to cellularly diffuse stimuli. The region of p65 implicated in synaptic enrichment bears little conservation across other Rel family subunit proteins and largely lacks previously characterized functional domains, indicating that these have either not yet been defined in other cell types or that the region has a unique functional significance in neurons. The region of p65 implicated in synaptic enrichment does include a Src homology 3 (SH3) poly-proline binding motif as well as an intrinsically disordered region predicted with high confidence (44). Mature dendritic spines are connected to the parent dendrite of neurons by relatively thin spine necks that can constrict cytoplasmic ionic and biochemical fluxes in response to incoming stimuli. Consequently, subcellular restriction of signaling pathways could confer value to co-compartmentalization of the NF-κB transcription factor at excitatory synapses in neurons. Evaluation of hippocampal pyramidal neurons lacking dendritic spine enrichment of NF-κB revealed that they had less mature dendritic spines and a reduced density of dendritic spines compared to wildtype hippocampal neurons (44). Interestingly, the p65 subunit of NF-κB is also reported to be enriched at the axon initial segment in cortical neurons, where it is proposed to be sequestered by binding to ankyrin G (46); it is not yet clear if these effects are selective only for the p65 subunit.

Broadly summarized, work from many labs indicates that neuronal NF-κB functions under normal physiological conditions to promote synapse growth and to enhance synaptic activity and enduring forms of plasticity. In addition to gene targets previously characterized in the immune and cancer fields, NF-κB has also been shown to regulate downstream targets with particular relevance for synaptic plasticity, including PSD-95, SPAR, PKA, nNOS, and growth factors, such as BDNF and IGF-2(18–21, 23, 24, 47–49). Functions in neuronal plasticity may underlie the requirements for NF-κB in behavioral readouts of cognition documented in many investigations, however some behavioral experiments have not utilized neuronal-specific manipulations of the NF-κB pathway so NF-κB in other cell types could participate in observed phenotypes. The enrichment of NF-κB at excitatory synapses and its activation by excitatory synaptic activity are also key to the unique roles of the NF-κB pathway in mammalian neurons.

## Glia

Glial cells within the CNS are comprised of four basic types: astrocytes, oligodendrocytes, microglia, and ependymal cells (epithelial lining which produce cerebral spinal fluid). For the purposes of this review, we will focus primarily on astrocytes and microglia. In contrast to neurons, NF-κB in glial cells has not been reported to be activated by excitatory neurotransmitters or under basal conditions. Reporter assays of NF-κB-dependent gene expression in primary glial cultures from normal murine cortex or in cryosections showed no evident NF-κB activity under basal conditions in glial cells that were identified through glial fibrillary acidic protein (GFAP) staining (50, 51). GFAP serves to mark many astrocytes and ependymal cells, as well as some oligodendrocytes and precursor cells in the mature CNS. Due to the limited basal NF-κB activity in glia, many studies have investigated the glial functions of NF-κB in settings of inflammation, injury or disease. As in neurons, the predominant activated form of NF-κB in mature glial cells appears to contain the RelA/p65 subunit, rather than utilizing non-cannonical signaling mediated by RelB:p52 heterodimers. However, evidence for the involvement of p52 and RelB has been reported in neural stem cells from the adult mouse nervous system following lymphotoxin β receptor-mediated signaling (52). RelB and p52 are also reported to function in driving tumor progression in glioma cell lines and is correlated with invasive potential (53). In addition, roles for RelB in reactive astrocytes (54) and microglia (55, 56) have been reported in the setting of chronic inflammation, such as can occur following brain injury or infection.

Microglia cells are the specialized population of resident macrophages in the CNS responsible for immune defense. Under normal conditions, these cells comprise an estimated 10–15% of all cells within the CNS. Brain microglia are typically in a resting state, but in the context of injury or disease they can become activated and aid in clearing cellular debris or innate immunity functions. Under chronic or extreme conditions this activation can lead to an overproduction of cytotoxic factors, such as excess nitric oxide, IL-1β and TNFα (57). In this setting, activation of NF-κB signaling pathways in microglia and consequent production of inflammatory mediators can exacerbate neuronal cell death. In primary cultures from mice with reduced microglial IKK activity (by conditional loss of IKKβ exon 3 in the myeloid lineage), the production of inflammatory mediators and hippocampal neuronal cell death in response to kainic acid exposure was reduced, in comparison to wildtype cultures with an intact microglial NF-κB pathway (58). NF-κB signaling in microglia may also play a role in the healthy brain by exerting homeostatic regulation of neuronal excitability and synaptic plasticity. Mice with selective depletion of IKKβ in microglial cells (by conditional loss of IKKβ exon 3 in the myeloid lineage), are reported to exhibit reduced brain expression of the NF-κB target genes, IL-1β, IL-6, and inducible NOS, and also display behavioral defects in hippocampal-dependent associative learning (22). In the same model system, *in-vitro* assays of plasticity, including long-term potentiation and excitatory field potentials, were consistent with the microglial NF-κB pathway participating in the down-regulation of neuronal excitability (22). This is an interesting contrast to the pro-excitation cis-regulatory role supported for NF-κB within neurons. Conditional deletion of an NF-κB regulatory protein, the A20 deubiquitinase, in microglia also supports roles for microglial NF-κB signaling in both neuronal homeostasis as well as in response to injury (59). Microglial deficiency in A20 resulted in increased numbers of microglia and an increase in synaptic excitation (59). Collectively, these studies are also consistent with the previously established roles of microglia in developmental and learning-associated synapse formation within the CNS (60, 61).

Astrocyte lineages are found throughout the CNS and have long been appreciated for their function in forming the blood brain barrier as well as signaling in the support and repair of neurons. While astrocytes are the most numerous and diverse glial cells with multiple astrocyte subtypes described, the understanding of astrocyte NF-κB function currently lacks this depth and is best characterized for astrocytes as a whole. Multiple studies have demonstrated that signaling through NF-κB in astrocytes contributes to pro-inflammatory responses following injury and that inhibition of NF-κB in astrocytes can promote functional recovery. For example, expression of a DN-IκBα driven by the GFAP promoter, has been shown to reduce cytokine expression, prevent damage to neurons and nerves, and to improve recovery after spinal cord or optic nerve injury (62–64). A pro-inflammatory role of glial NF-κB is also well-documented in disease settings, several of which are discussed below. Astrocytic NF-κB has also been shown to have roles apart from promoting the expression of pro-inflammatory genes. In the healthy CNS, astrocytes play a critical role in effective termination of excitatory signals by clearing glutamate released from synapses in part through the glutamate transporter-1 (GLT-1). The dynamic induction of astrocyte GLT-1, which is dependent upon the presence of neurons and neuronal activity-dependent activation of NF-κB in astrocytes, has been shown to be largely ablated by inhibition of astrocyte NF-κB using DN-IκB expression in culture (65). Critical NF-κB regulatory sites on the GLT-1 gene were identified (65). Recently, the astrocyte NF-κB pathway has also been implicated in the central control of metabolism, including regulation of blood sugar, blood pressure, and body weight (66). Astrocytes undergo dynamic structural plasticity of their processes, which can be modulated in the hypothalamus in response to metabolic information regarding the fed or unfed state (66). Mice expressing CA-IKKβ under control of the GFAP promoter were found to have impaired astrocyte plasticity with sustained astrocyte process shortening in the hypothalamic region of the brain, a phenotype also observed with chronic overnutrition. While transient modulation of this astrocyte NF-κB signaling pathway could participate in metabolic responses, the experimental setting of chronic CA-IKKβ expression was observed to lead to metabolic disease including glucose intolerance and obesity.

It should be noted that a general caveat to many studies that use genetic manipulations to investigate glial-specific roles of the NF-κB pathway is the lack of suitable promoters and identifying markers for glial cells. GFAP is a common marker used to identify astrocytes and to drive manipulations in glial cells, but GFAP has also been shown to be present in certain neuronal subtypes as well as in some precursor cells (67). For example, a transgenic mouse line expressing the DN-IκB under control of the GFAP promoter, displays expression in precursor cells and a deficit in adult neurogenesis (68), as well as expression in adult astroglial cells which complicates determining the origins of observed learning and memory deficits (69). GFAP-promoter driven expression of DN-IκB in cultured neural stem cells is reported to promote glial lineage differentiation at the expense of neuronal lineage differentiation (52). Recently a new astrocyte-selective marker has been identified, Aldh1I1, which is reported to show little or no detection in neuronal populations or precursors (67). In the future, fruitful investigations of NF-κB function in glia will hopefully have the opportunity to make use of increasingly selective tools for glial subtype expression, such as the Aldh1I1 promoter for astrocytes.

### Disease

As in the immune system, numerous roles for NF-κB in disorders of the CNS have been documented. A chief controversy has been whether NF-κB plays primarily a protective effect on neuronal health, or whether its pro-inflammatory actions exacerbate neuronal apoptosis in settings of CNS disease. Early evidence using cell-type specific expression of the dominant negative IκB (DN-IκB) to selectively inhibit NF-κB in neurons indicated that NF-κB within neurons played an anti-apoptotic role (16). This is consistent with evidence that, in addition to regulating immune and inflammatory genes, NF-κB also regulates the expression of growth factors as well as genes that antagonize cell death. NF-κB has been shown to regulate anti-apoptotic genes including caspase inhibitors, TNF-receptor associated factors, TRAF1 and TRAF2, and the Bcl-2 family (Bcl-2, Bcl-x_L_, Bfl-1) (70–75). Cis-regulation of anti-apoptotic genes in neurons can confer resistance to death-inducing signals under adverse conditions and enhance survival in response to growth factors (75–78). With some exceptions and subunit dependence [as reviewed elsewhere (79)], substantial evidence now supports an anti-apoptotic role for NF-κB within neurons while prolonged NF-κB activation in reactive glial cells, has been associated with detrimental outcomes, inflammation, and neuronal cell death. Overall In this section, we will focus on selected disease examples to highlight differential roles of NF-κB in glia vs. neurons in disorders of the CNS. Table 1 shows functions of NF-κB in the CNS and how these functions are altered in disease states.

**Table 1:** Summary of NF-κB roles in neurons and glia, both in normal physiology and disease.

**Cell type**	**Overview of NF-κB functions**	
	**Normal physiology**	**Disease**
Neuron	^*^ Synaptic plasticity ^*^ Learning and memory ^*^ Synapse to nuclear communication ^*^ Developmental growth and survival in response to trophic cues	^*^ Aberrant synapse to nuclear communication ^*^ Protective from death-inducing signals associated with injury or inflammatory mediators ^*^ Protective against apoptosis in neurodegenerative models
Glia	^*^ Immune response ^*^ Injury response ^*^ Glutamate clearance ^*^ Central control of metabolism	^*^ Chronic NF-κB activation elevates neuroinflammation and neuronal cell death ^*^ Prolonged NF-κB induction increases activated microglia ^*^ c-Rel loss increases microglial activation

*The pleiotropic functions of the NF-κB signaling pathway coupled with the cellular diversity of the nervous system mean that this table reflects generalizations, while more specific details are in the text of this review*.

## Alzheimer's Disease

Alzheimer's disease (AD) is an age-related neurodegenerative disease characterized by progressive loss of memory and other cognitive functions, changes in behavior, difficulty completing basic tasks, and confusion. An accumulation of amyloid-β (Aβ) plaques and neurofibrillary tangles in the brain, as well as neuroinflammation and vascular alterations, are hallmarks of AD. The link of plaques and neurofibrillary tangles to disease pathology remains uncertain and is an area of active investigation. Aberrant neural network activity, synaptic dysfunction and synapse loss correlate strongly to decline in cognitive function and neurodegeneration, but the molecular mechanisms are not fully understood and some have been difficult to recapitulate in mouse models of the disease (80). Genetic studies link multiple genes to AD development; including amyloid precursor protein (APP), Presenilin 1 (PSEN1), Presenilin 2 (PSEN2), apolipoprotein E (ApoE), and Triggering Receptor Expression on Myeloid cells 2 (TREM2), along with others (81). Promoter analysis and functional studies link expression of each of these genes to regulation by NF-κB (82–85). In some cases, products from AD-associated genes, such as presenilin 1, have also been shown to mediate reciprocal activation of NF-κB (RelA/p65 containing dimers) in putative pro-inflammatory cascades (86).

A prominent role for neuroinflammation associated with neurodegenerative changes has been documented, but the complexity of immune cell types in the brain has contributed to conflicting reports attributing the aberrant inflammation to either systemic immunity, brain-recruited monocytes, or brain-resident microglia. It has also been debated whether the recruitment of microglia might be beneficial (albeit insufficient) in combatting neurodegenerative processes in AD, or whether microglial activation might be a contributing factor to neurodegeneration. TREM family proteins are part of a neuroinflammatory cascade, and evidence supports NF-κB as a central player governing expression of both TREM1 and TREM2(84). Microglial activation driven by NF-κB (RelA) and cytokine signaling is reported in data from microglia isolated from a tau transgenic mouse model (rTg4510) of AD where downstream differential expression of TREM2 and APOE was also observed (87). In microglia, TREM2 functions as a surface receptor required for responses associated with activation, including survival, proliferation, and phagocytosis. A hypomorphic variant of TREM2 is associated with elevated risk of late-onset AD in humans while TREM2 loss of function mutations as associated with dementia, and mice deficient in TREM2 develop AD-associated pathologies (88, 89). Recently, single cell sequencing approaches have allowed finer resolution of the immune cell heterogeneity in AD and have shed light on a potential protective microglial type associated with neurodegeneration. This research revealed that a TREM2-dependent program activated in a unique disease-associated microglia (DAM) type was associated with restricting the development of AD and was beneficial to mitigating the disease likely through phagocytosis (90). Findings of this study are in-line with a second recent report, from different investigators, which showed that TREM2 intracellular signaling functions to maintain the metabolic fitness and phagocytic responses of microglia operating to defend the brain in AD (91, 92). Since microglia are active participants in the formation, remodeling, and elimination of synapses, this research may also shed light on the mechanisms which underlie synapse loss in AD (93, 94). This research also raises the issue of whether NF-κB-dependent regulation of microglia TREM2 expression might also play an as-yet unexplored role in the synaptic plasticity associated with learning and memory.

The role of NF-κB in AD was recently covered more broadly in a dedicated review (6) which discussed the regulation of NF-κB by Aβ as well as giving a comprehensive overview of NF-κB targets with potential implications in AD development or cognitive symptoms, including CREB, MnSOD, CAMKII, and PSD95. NF-κB has also been linked to regulation of ApoE, of which the ApoE4 variant is the strongest genetic risk factor for development of late onset AD, while ApoE3 is neutral and ApoE2 is protective. Gene promoter analysis identified NF-κB binding sites upstream of the ApoE transcription initiation region (95) and characterization of ApoE4 transcriptional regulation through the use of luciferase reporter assays in glial cells stimulated with Aβ confirmed functional regulation of expression through NF-κB signaling (82). Further studies are needed to determine the extent to which NF-κB may regulate ApoE4 and other ApoE variants in the brain and whether these signaling pathways are impacted in AD. The dual functions of NF-κB in cognitive processes and inflammatory cascades have highlighted interest in NF-κB as a therapeutic target for early intervention in treatment of AD. Interestingly, not only genetic but also environmental risk factors for AD, as well as protective factors, such as diet, anti-inflammatory medications, and exercise, show correlative relationships to NF-κB (96). Aging, the most significant risk factor for AD, is also associated with elevated levels of brain NF-κB activation and tissue-specific inflammation with relevance to AD and other neurodegenerative processes (96). While putative cell-type specific roles for NF-κB in microglia have emerged with its function in TREM2 regulation, continued exploration will be needed to explore whether other risk factors exhibit cell-type specific roles for NF-κB in AD.

## Huntington's Disease

Huntington's disease (HD) is an inherited autosomal dominant neurodegenerative disorder characterized by changes in mood and a decrease in coordination and mental abilities, and is progressive and fatal. HD is caused by mutations expanding the CAG triplet repeat region of the Huntington gene that encodes a polyglutamine tract (polyQ) in the amino-terminus of the Huntingtin protein (HTT). Normal HTT has <26 repeats, while the mutated version leading to disease can typically have >35 repeats, with a higher repeat number correlated to increased severity and an earlier disease onset. Research from several investigators suggests that the polyglutamine expansion may alter the conformation of HTT protein (97). HTT is expressed throughout the body, but the normal function of HTT and why mutated HTT is most disruptive to neurons remain incompletely understood. Normal HTT has been shown to interact with various neuronal proteins, including both the p50 and p65 subunits of NF-κB (45, 98). HTT has also been shown to enhance intracellular transport through interaction with the cytoplasmic dynein molecular motor (99, 100), and this function of HTT is disrupted by the polyQ expansion associated with HD (100, 101). Dynein and the dynactin complex move cargo toward the minus-ends of microtubules, facilitating retrograde transport in neuronal dendrites, and nuclear transport of activated NF-κB following stimulation requires dynein and the dynactin complex (38, 40). Interestingly, HTT is enriched at the post-synaptic density of neuronal synapses, along with the p50 and p65 NF-κB subunits, and has been reported to preferentially associate with activated NF-κB and to enhance the movement of p65-containing NF-κB dimers out of dendritic spines. The polyQ expansion of HTT associated with HD impairs the enrichment of HTT in the PSD and reduces the movement out of dendritic spines and nuclear accumulation of NF-κB (45). This work suggests that aberrant synapse-to-nucleus transport of NF-κB in neurons could participate in the etiology of HD.

While the majority of studies have linked NF-κB to HD specifically in neuronal cell types, mutant HTT has also been found to affect neuroinflammation, which could suggest a role in glia. Astrocytes from the caudate nucleus brain region of human patients with HD and from the cortex of a mouse model of HD exhibit increased activation of NF-κB (nuclear localization of RelA/p65) (102). Under basal conditions, the increased activation of NF-κB only occurred in astrocytes and not neurons or microglia. The increased activation of NF-κB was reported to be due to elevated astrocyte IKK activity which agreed with a previous study showing higher IKK activities in the brains of a mouse model of HD (103). Blockade of IKK alleviated neurotoxicity caused by the HD astrocytes and ameliorated symptoms of HD (102). NF-κB has also been shown to regulate HTT at the transcription level; analysis of the HTT promoter identified an NF-κB binding site that regulates HTT transcription, as well as a SNP in this binding site which impaired NF-κB binding and lowered HTT transcription (104). Importantly, this SNP was shown to impact development of HD in an allele-specific manner; when the SNP was present on the HTT mutant allele a protective effect of delayed onset was observed in HD patients while early onset HD was associated with the presence of the SNP on the wildtype HTT allele. While this study was primarily conducted at the genomic, rather than cell-type specific, level, effects of the NF-κB binding site and SNP in the HTT promoter were validated in ST14A cells, which are derived from the striatal brain region and display features of medium spiny neurons (104). These findings highlight the importance of the NF-κB pathway in regulating HTT gene expression and progression to HD.

## Amyotrophic Lateral Sclerosis

Amyotrophic lateral sclerosis (ALS) is caused by death of motor neurons which leads to worsening ability to move voluntary muscles and eventually leading to difficulty speaking and breathing. While most cases of ALS are sporadic, about 10% of ALS cases are inherited. Aberrant expression or mutation of multiple genes have been associated with ALS, with chromosome 9 open reading frame 72 (C9ORF72), superoxide dismutase-1 (SOD1), NIMA-related kinase 1 (NEK1), FUS RNA binding protein (FUS), and TAR DNA-binding protein (TDP-43) collectively being amongst the most common genes in the familial fraction of ALS cases. NF-κB has been shown to be involved in the regulation or interaction with several of these genes. An NF-κB binding site identified in the promoter of human SOD1 was reported to mediate increases in SOD1 levels in response to PI3K/Akt signaling (105). The p65 subunit of NF-κB has been shown to undergo protein-protein interaction with TDP-43, an association that is increased in ALS. TDP is proposed to play a co-activator role for NF-κB and inhibition of NF-κB reduces inflammation and neuron death (106). Mutations in optineurin (OPTN) have also been shown to be associated with ALS. While wildtype OPTN has been shown to negatively regulate TNFα-induced activation of NF-κB, familial ALS-associated mutations in OPTN abolish this inhibition of NF-κB activation (107).

Neuroinflammation and the activation of microglia are hallmarks of ALS. Activated NF-κB in glial cells with both inherited and sporadic forms of ALS has been demonstrated by immunohistochemistry (106). Increased microglial activation of NF-κB (by EMSA and phospho-p65 immunoblot) in spinal cord of both human patients with ALS and in the SOD1-G93A mouse model of ALS has been shown to parallel disease progression (108). In this mouse model, the death of motor neurons could be rescued through selective NF-κB inhibition (IKKβ deficiency or DN-IκB expression, using colony stimulating factor receptor (CSF-1R) promoter driver which is microglia selective within the post-natal mouse brain) in microglial cells, while NF-κB inhibition in the astrocyte glial subtype was without effect (108). Recent work suggests that NF-κB activation in astrocytes may also play a role in ALS, in part by regulating the proliferation and immune response in microglia (109), albeit using the GFAP promoter which is also active in neural precursors to drive CA-IKKβ. While astrocyte NF-κB activation and corresponding microglial proliferation was shown to be neuroprotective during the pre-symptomatic phase, astrocyte NF-κB activation in later symptomatic phases worsened disease progression by increasing pro-inflammatory microglial activation (109); it should be noted for inferences from this study that the GFAP promoter which is also active in neural precursors was used to drive CA-IKKβ. In summary, in addition to the targeted motor neurons, NF-κB activation in non-neuronal cells plays a crucial role in the pathogenesis of ALS.

## Parkinson's Disease

Parkinson's disease (PD) is a progressive neurodegenerative disease affecting the motor system and frequently eventually accompanied by dementia. Early symptoms can include tremor, muscle rigidity, slowed movement, and difficulty walking and can also include mental and emotional problems and psychosis. The causes of PD are not fully understood but genetic and environmental factors, as well as inflammatory mechanisms are associated with the disease. Symptoms are thought to arise primarily due to death of dopaminergic neurons in the midbrain substantia nigra, which has been attributed to cellular disturbances including protein aggregation, ER stress, mishandling of calcium, and mitochondrial dysfunction (110). Mutations in multiple genes have been linked to PD, including alpha-synuclein (SNCA), leucine-rich repeat kinase 2 (LRRK2), glucosylceramidase beta (GBA), Parkin, and PTEN-induced kinase 1 (PINK1); several studies have suggested NF-κB as a therapeutic target for PD arising from multiple genetic etiologies. Consistent with the pro-inflammatory state, activation of NF-κB by nuclear translocation is observed in post-mortem brains of patients diagnosed with PD and in animal models of PD (111, 112). While nuclear p65 has been observed in neurons as well as astrocytes in tissue from PD patients, whether activated NF-κB occurs primarily in neurons or glia in mouse models of PD may depend upon the model under study. Toxicity induced by 1-methyl-4-phenyl−1,2,3,6-tetrahydropyridine (MPTP) has been employed to produce dopaminergic neuron death in mice for the study of PD. In the MPTP-toxicity model, the induction of pro-inflammatory astrocytes accompanied by NF-κB activation in astrocytes is reported (111). In this context, IKK inhibition by injection of a cell-permeant nemo-binding domain (NBD) peptide reduced pro-inflammatory astrocytes as well as substantia nigra dopaminergic neuron cell death in response to MPTP (111).

Several publications have supported a role for activation of c-Rel-containing dimers in conferring protection from neurodegenerative-associated stimuli through induction of anti-apoptotic genes (113–115). Mice globally deficient in the c-Rel subunit exhibit a significant late-onset loss of dopaminergic neurons and dopaminergic synaptic terminals in the substantia nigra of aged mice (18 months) as well as a deficiency in motor activity as compared to wild type mice (114), consistent with a PD phenotype. Lewy bodies and eosinophilic inclusions containing α-synuclein are characteristic findings in brains from individuals diagnosed with PD. Aged c-Rel^−/−^ mice were observed to have elevated α-synuclein-positive inclusions that were selectively located within the dopaminergic neuron population, as opposed to either other neuronal types or glia. While no change in activated astrocytes was observed, an increase in numbers of activated microglia were observed by immunostaining in the brains of aged c-Rel-deficient compared to wildtype mice (114).

NF-κB has also been linked with proteins known to be involved in PD. Epistasis studies examining signaling of PD-associated genes in drosophila first suggested that PINK1 may act upstream of Parkin in a common pathway (116–118). Studies of the molecular mechanisms of neurodegeneration triggered by autosomal recessive mutations of either PINK1 or Parkin have demonstrated a role for NF-κB (119, 120). The expression of wildtype Parkin in a neuroblastoma cell line was shown to activate NF-κB through IKK, using luciferase reporter and gel shift assays, while expression of Parkin harboring pathogenic mutations associated with PD exhibited reduced capacity to activate NF-κB (119). Loss of Parkin function in either the neuroblastoma cell line, or in fibroblasts from PD patients with Parkin mutations, inhibited activation of the NF-κB pathway in response to cellular stressors (119). Mechanistically, parkin is reported to possess E3 ligase activity with Parkin activation mediating K63-linked polyubiquitination of IKKy and consequent NF-κB activation and protection against apoptosis (120). In addition, a brain-specific tripartite motif protein (TRIM9) with lowered levels in post-mortem brains of PD patients (121), was recently reported to function as an inhibitor of NF-κB activation by blocking IκBα degradation to restrict neuroinflammation (122). As TRIM9 manipulations have not been made in a cell-type selective manner, it is not yet clear whether the relevant TRIM9 functions in resolving NF-κB activation are occurring in neurons or glia (122). Collectively, research to date supports a role for deficiencies of NF-κB activation in reducing neuroprotection and neuron survival in association with PD. However, while NF-κB activation in neurons is clearly shown to be a protective feature disrupted by PD in multiple studies, consensus is lacking regarding the relative importance of NF-κB signaling in neurons compared to glial cells for pathology of PD.

## Conclusion

Dysregulation of NF-κB activation and NF-κB-dependent gene expression have been implicated in a host of brain disorders. In this review, we have selected several as illustrative examples in considering cell and NF-κB subunit-type specific roles for brain NF-κB. In conclusion, NF-κB is widely expressed in all cell types in the brain but its activation or deficiency in different cell types reveals different functions and consequences both on cell-autonomous and non-cell autonomous levels (Table 2 summarizes cell-type specific studies from this review). NF-κB functions in neurons under basal conditions to maintain neuronal health, synapse growth and plasticity-related functions, and under disease conditions, upregulation in neurons is associated with neuroprotective outcomes. While NF-κB is reported to have little basal activity in glia, under certain conditions NF-κB-dependent gene expression in glial subtypes can have beneficial outcomes in maintaining brain health through immune response and neuronal maintenance (such as in DAM), but chronic or excessive glial activation of NF-κB has been shown to be neurotoxic. The NF-κB subunits, RelA/p65, p50, and c-Rel have well-documented importance in healthy physiological responses in neurons and behavioral assays of cognition. The specific NF-κB subunits of functional importance in glial cells are for the most part less well-defined than in neurons, but RelA/p65 has been implicated in expression of inflammatory mediators in microglia. To date, roles for RelB and p52 reported in the literature appear mostly circumscribed to instances of pluripotent neural stem cells, transformed cells, such as glioma/glioblastoma, and several reports of expression in activated microglia. While initial characterization suggested that RelB expression was largely restricted to hematopoietic cells, examination of the nervous system was not specifically reported (123). However, the updated Human Protein Atlas (www.proteinatlas.org, based on commercially available antibodies) reports medium to high levels of RelB protein in human brain neurons in both the cortex and hippocampus. It remains unclear whether this potential discrepancy reflects as yet unknown functions for RelB and p52 in the healthy nervous system, or a difference between mouse and human nervous system expression.

**Table 2:** A reference categorizing primary literature cited in this review according to the specificity of the brain cell type in which NF-κB is investigated: neurons, microglia, or astrocytes.

**References**	**Title**	**First author**	**Year**
**NEURONS**			
(7)	NF-kappaB p50-deficient mice show reduced anxiety-like behaviors in tests of exploratory drive and anxiety	Kassed	2004
(8)	Lack of NF-kappaB p50 exacerbates degeneration of hippocampal neurons after chemical exposure and impairs learning	Kassed	2002
(9)	NF-kappaB p50 subunit knockout impairs late LTP and alters long term memory in the mouse hippocampus	Oikawa	2012
(10)	c-Rel, an NF-kappaB family transcription factor, is required for hippocampal long-term synaptic plasticity and memory formation	Ahn	2008
(11)	A bioinformatics analysis of memory consolidation reveals involvement of the transcription factor c-rel	Levenson	2004
(12)	Regulation of nuclear factor kappaB in the hippocampus by group I metabotropic glutamate receptors	O'Riordan	2006
(13)	NF-kappa B functions in synaptic signaling and behavior	Meffert	2003
(14)	Late-Life Environmental Enrichment Induces Acetylation Events and Nuclear Factor kappaB-Dependent Regulations in the Hippocampus of Aged Rats Showing Improved Plasticity and Learning	Neidl	2016
(15)	The IkappaB kinase regulates chromatin structure during reconsolidation of conditioned fear memories	Lubin	2007
(16)	Forebrain-specific neuronal inhibition of nuclear factor-kappaB activity leads to loss of neuroprotection	Fridmacher	2003
(17)	NF-kappaB/Rel regulates inhibitory and excitatory neuronal function and synaptic plasticity	O'Mahony	2006
(18)	NF-kappaB transcription factor role in consolidation and reconsolidation of persistent memories	de la Fuente	2015
(22)	Differential contributions of microglial and neuronal IKKbeta to synaptic plasticity and associative learning in alert behaving mice	Kyrargyri	2015
(23)	A requirement for nuclear factor-kappaB in developmental and plasticity-associated synaptogenesis	Boersma	2011
(24)	Opposing action of nuclear factor kappaB and Polo-like kinases determines a homeostatic end point for excitatory synaptic adaptation	Mihalas	2013
(25)	Stimulation of ionotropic glutamate receptors activates transcription factor NF-kappa B in primary neurons	Kaltschmidt	1995
(26)	Synaptic activation of NF-kappa B by glutamate in cerebellar granule neurons *in vitro*	Guerrini	1995
(27)	Glutamate-dependent activation of NF-kappaB during mouse cerebellum development	Guerrini	1997
(28)	Glutamate promotes NF-kappaB pathway in primary astrocytes: protective effects of IRFI 016, a synthetic vitamin E analog	Caccamo	2005
(29)	Essential role for epidermal growth factor receptor in glutamate receptor signaling to NF-kappaB	Sitcheran	2008
(31)	From calcium to NF-kappa B signaling pathways in neurons	Lilienbaum	2003
(33)	Kainate receptors activate NF-kappaB via MAP kinase in striatal neurones	Cruise	2000
(37)	Glutamate-induced NFkappaB activation in the retina	Fan	2009
(38)	Stimulated nuclear translocation of NF-kappaB and shuttling differentially depend on dynein and the dynactin complex	Shrum	2009
(39)	Single-particle tracking uncovers dynamics of glutamate-induced retrograde transport of NF-kappaB p65 in living neurons	Widera	2016
(40)	Transcription factor NF-kappaB is transported to the nucleus via cytoplasmic dynein/dynactin motor complex in hippocampal neurons	Mikenberg	2007
(41)	Brain synapses contain inducible forms of the transcription factor NF- kappa B	Kaltschmidt	1993
(42)	Gene expression of the transcription factor NF-kappa B in hippocampus: regulation by synaptic activity	Meberg	1996
(43)	Hippocampal dynamics of synaptic NF-kappa B during inhibitory avoidance long-term memory consolidation in mice	Salles	2015
(44)	Targeting of NF-kappaB to Dendritic Spines Is Required for Synaptic Signaling and Spine Development	Dresselhaus	2018
(45)	The Huntington's disease mutation impairs Huntingtin's role in the transport of NF-kappaB from the synapse to the nucleus	Marcora	2010
(46)	NF-kappaB regulates neuronal ankyrin-G via a negative feedback loop	Konig	2017
(47)	IkappaB kinase/nuclear factor kappaB-dependent insulin-like growth factor 2 (Igf2) expression regulates synapse formation and spine maturation via Igf2 receptor signaling	Schmeisser	2012
(48)	NF-kappaB regulates spatial memory formation and synaptic plasticity through protein kinase A/CREB signaling	Kaltschmidt	2006
(49)	Role of p300 in regulating neuronal nitric oxide synthase gene expression through nuclear factor-kappaB-mediated way in neuronal cells	Li	2013
(50)	Constitutive nuclear factor-kappa B activity is required for central neuron survival	Bhakar	2002
(51)	NF-kappaB activity in transgenic mice: developmental regulation and tissue specificity	Schmidt-Ullrich	1996
(52)	Lymphotoxin beta receptor-mediated NFkappaB signaling promotes glial lineage differentiation and inhibits neuronal lineage differentiation in mouse brain neural stem/progenitor cells	Xiao	2018
(76)	NFkappaB activation is required for the neuroprotective effects of pigment epithelium-derived factor (PEDF) on cerebellar granule neurons	Yabe	2001
(77)	The canonical nuclear factor-kappaB pathway regulates cell survival in a developmental model of spinal cord motoneurons	Mincheva	2011
(98)	The predominantly HEAT-like motif structure of huntingtin and its association and coincident nuclear entry with dorsal, an NF-kB/Rel/dorsal family transcription factor	Takano	2002
(104)	A SNP in the HTT promoter alters NF-kappaB binding and is a bidirectional genetic modifier of Huntington disease	Becanovic	2015
(105)	Regulation of Cu/Zn-superoxide dismutase expression via the phosphatidylinositol 3 kinase/Akt pathway and nuclear factor-kappaB	Rojo	2004
(106)	Deregulation of TDP-43 in amyotrophic lateral sclerosis triggers nuclear factor kappaB-mediated pathogenic pathways	Swarup	2011
(112)	Nuclear translocation of NF-kappaB is increased in dopaminergic neurons of patients with parkinson disease	Hunot	1997
(113)	NF-kappaB factor c-Rel mediates neuroprotection elicited by mGlu5 receptor agonists against amyloid beta-peptide toxicity	Pizzi	2005
(114)	Late-onset Parkinsonism in NFkappaB/c-Rel-deficient mice	Baiguera	2012
(119)	Parkin mediates neuroprotection through activation of IkappaB kinase/nuclear factor-kappaB signaling	Henn	2007
(120)	Phosphorylation of parkin by Parkinson disease-linked kinase PINK1 activates parkin E3 ligase function and NF-kappaB signaling	Sha	2010
(122)	TRIM9-Mediated Resolution of Neuroinflammation Confers Neuroprotection upon Ischemic Stroke in Mice	Zeng	2019
**MICROGLIA**			
(22)	Differential contributions of microglial and neuronal IKKbeta to synaptic plasticity and associative learning in alert behaving mice	Kyrargyri	2015
(55)	Regulation of inflammatory responses by neuregulin-1 in brain ischemia and microglial cells *in vitro* involves the NF-kappa B pathway	Simmons	2016
(56)	Nuclear factor-kappa B family member RelB inhibits human immunodeficiency virus-1 Tat-induced tumor necrosis factor-alpha production	Kiebala	2010
(58)	Role of microglial IKKbeta in kainic acid-induced hippocampal neuronal cell death	Cho	2008
(59)	A20 critically controls microglia activation and inhibits inflammasome-dependent neuroinflammation	Voet	2018
(60)	Microglia promote learning-dependent synapse formation through brain-derived neurotrophic factor	Parkhurst	2013
(61)	Microglia contact induces synapse formation in developing somatosensory cortex	Miyamoto	2016
(84)	Divergent Neuroinflammatory Regulation of Microglial TREM Expression and Involvement of NF-kappaB	Owens	2017
(87)	Genome-wide RNAseq study of the molecular mechanisms underlying microglia activation in response to pathological tau perturbation in the rTg4510 tau transgenic animal model	Wang	2018
(106)	Deregulation of TDP-43 in amyotrophic lateral sclerosis triggers nuclear factor kappaB-mediated pathogenic pathways	Swarup	2011
(108)	Microglia induce motor neuron death via the classical NF-kappaB pathway in amyotrophic lateral sclerosis	Frakes	2014
(109)	NF-kappaB activation in astrocytes drives a stage-specific beneficial neuroimmunological response in ALS	Ouali Alami	2018
**ASTROCYTES**			
(52)	Lymphotoxin beta receptor-mediated NFkappaB signaling promotes glial lineage differentiation and inhibits neuronal lineage differentiation in mouse brain neural stem/progenitor cells	Xiao	2018
(54)	RelB controls adaptive responses of astrocytes during sterile inflammation	Gupta	2019
(62)	Inhibition of astroglial nuclear factor kappaB reduces inflammation and improves functional recovery after spinal cord injury	Brambilla	2005
(63)	Transgenic inhibition of astroglial NF-kappaB protects from optic nerve damage and retinal ganglion cell loss in experimental optic neuritis	Brambilla	2012
(64)	Transgenic inhibition of astroglial NF-kappa B leads to increased axonal sparing and sprouting following spinal cord injury	Brambilla	2009
(65)	Nuclear factor-kappaB contributes to neuron-dependent induction of glutamate transporter-1 expression in astrocytes	Ghosh	2011
(66)	Astrocytic Process Plasticity and IKKbeta/NF-kappaB in Central Control of Blood Glucose, Blood Pressure, and Body Weight	Zhang	2017
(69)	Astroglial nuclear factor-kappaB regulates learning and memory and synaptic plasticity in female mice	Bracchi-Ricard	2008
(82)	NF-(kappa)B mediates amyloid beta peptide-stimulated activity of the human apolipoprotein E gene promoter in human astroglial cells	Du	2005
(102)	A critical role of astrocyte-mediated nuclear factor-kappaB-dependent inflammation in Huntington's disease	Hsiao	2013
(103)	Activation of the IkappaB kinase complex and nuclear factor-kappaB contributes to mutant huntingtin neurotoxicity	Khoshnan	2004
(106)	Deregulation of TDP-43 in amyotrophic lateral sclerosis triggers nuclear factor kappaB-mediated pathogenic pathways	Swarup	2011
(109)	NF-kappaB activation in astrocytes drives a stage-specific beneficial neuroimmunological response in ALS	Ouali Alami	2018
(111)	Selective inhibition of NF-kappaB activation prevents dopaminergic neuronal loss in a mouse model of Parkinson's disease	Ghosh	2007

*Only references implicating or investigating specific cell types are included. Full reference information can be found in the bibliography of this article*.

Throughout this review, studies discussed as indicating cell-type specific roles for NF-κB are carried out generally by gene or pathway manipulation of NF-κB in a cell-type selective manner. In the setting of isolated cultured cell types, this type of manipulation can give rise to knowledge of cell-autonomous effects, such as RelA/p65-regulated growth of dendritic spines in hippocampal neurons (23). However, it should be noted that the outcomes (e.g., cognitive, behavioral, or neurodegenerative) observed from cell-type selective manipulations of NF-κB pathways can rarely be assigned as wholly cell-autonomous when assayed in the intact brain tissue or nervous system. Generating data to make this type of assignment would require extensive comparative NF-κB pathway manipulations in diverse cell-types, which has not been conducted in the nervous system to our knowledge. Given the current understanding of the complex interplay between the different cellular constituents, both in healthy cognitive processes and in disease, it is perhaps unlikely that a cell-type selective manipulation is capable of generating a purely cell-autonomous response in the intact nervous system. Nonetheless, cell-type selective initial manipulations of NF-κB can drive distinct outcomes in the intact nervous system and the current dearth of studies using spatially or temporally selective manipulations to target NF-κB subunits or NF-κB activation pathways represents a significant barrier to our understanding of NF-κB function in the nervous system. Elaborating our knowledge regarding specificity of NF-κB function in the CNS is an investment that can yield insights to the pleiotropic functions of NF-κB in healthy cognitive function and disease conditions.

## Author Contributions

All authors listed have made a substantial, direct and intellectual contribution to the work, and approved it for publication.

### Conflict of Interest Statement

The authors declare that the research was conducted in the absence of any commercial or financial relationships that could be construed as a potential conflict of interest.
